# Zwangsmaßnahmen in der Psychiatrie: praktische Konsequenzen ethischer Aspekte

**DOI:** 10.1007/s00115-020-00998-7

**Published:** 2020-09-30

**Authors:** Hanfried Helmchen

**Affiliations:** Klinik für Psychiatrie und Psychotherapie, CBF, Charité – Universitätsmedizin Berlin, Hindenburgdamm 30, 12200 Berlin, Deutschland

**Keywords:** Vermeidung von Zwang, Selbstbestimmungsfähigkeit, Sensibilisierung gegen Zwang, Kontrolle von Zwang, Entscheidungsassistenz, Prevention of coercion, Capacity of self-determination, Sensitization against coercion, Control of coercion, Assistance of decision

## Abstract

Im Rahmen der rechtlichen und ethischen Voraussetzungen und Verfahren der *Anwendung* von Zwang in der Psychiatrie wird die Perspektive der *Abwendung*, also die Prävention zur Vermeidung von Zwang beleuchtet. Zwang ist ausschließlich bei Selbstbestimmungsunfähigkeit und unmittelbarer konkreter Lebensgefährdung zulässig. Praktische Schwierigkeiten bei der Feststellung dieser Kriterien werden kasuistisch illustriert. Verfahren zur Vermeidung von Zwang (Vorausverfügungen, vertrauensbildende Maßnahmen, Erkennung von Kontexteinflüssen, Sensibilisierung gegenüber Zwang) zielen ebenso wie die Präzisierung von Indikation und Kontrolle der Anwendung von Zwang und ein Einstellungswandel hin zur Entscheidungsassistenz darauf, die Respektierung des Selbstbestimmungsrechtes des psychisch Kranken zu stärken – und damit den Konflikt des Psychiaters zwischen Fürsorgepflicht und Respektierung des Selbstbestimmungsrechtes des Patienten in ein faires Gleichgewicht zu bringen.

## Abstract (en)

Within the framework of the legal and ethical requirements of the application of coercion in psychiatry, the perspective of its prevention is discussed. Coercion is permissible exclusively in cases of incapability of self-determination and immediate specific danger for the patient’s physical integrity or life. Practical difficulties in assessing these criteria are illustrated by means of case examples. Preventive procedures for avoidance of coercion (advance directives, trust-building procedures, recognition of influence of context, sensitization against coercion) as well as clarification of indications and control of the application of coercion and a change of attitude toward assistance of decision aim at strengthening respect for the right to self-determination of the mentally ill and thereby to fairly balance the psychiatrist’s conflict between responsibility for care and respect for the right of self-determination of the patient.

Handeln im besten Interesse des Patienten und Schaden von ihm fernzuhalten sind ethische Prinzipien, zu denen sich Ärzte seit alters her selbst verpflichtet haben. Unter dem Begriff der Fürsorgepflicht sind sie heute auch rechtlich gefasst. Damit sind Psychiater als Ärzte verpflichtet, psychisch Kranken, die der Fürsorge bedürfen, kompetent zu helfen. Diese psychiatrische Hilfe zielt darauf, im objektiv „besten Interesse“ des Patienten zu handeln, also all jenes indizierte, d. h. evidenz- und erfahrungsbasierte Wissen einzusetzen, das das subjektive Leiden und gefährliche Verhaltensstörungen des Patienten bestmöglich mildert oder beseitigt und sein subjektives „Wohlbefinden“ fördert. Nicht immer jedoch ist ein Patient mit den psychiatrischen Maßnahmen einverstanden, sieht sich manchmal auch gar nicht als krank und behandlungsbedürftig an oder lehnt die psychiatrische Intervention rundheraus ab.

Dies wird dann zu einem schwerwiegenden Problem, wenn psychische Krankheit das Leben des Kranken gefährdet; und noch komplizierter kann es werden, wenn das Leben anderer Personen bedroht ist, weil dann auch die Gesellschaft über ihre Polizei reagiert – denn der Schutz des Lebens ist eine grundgesetzlich bestimmte Aufgabe des Staates (GG 2.2).^1^ Dieses Grundgesetz schützt aber ebenso die Menschenrechte (GG 1.2) und damit auch das Menschenrecht der Selbstbestimmung als „Recht auf die freie Entfaltung seiner Persönlichkeit“ (GG 2.1) [1]. Wenn nun ein Mensch in missbräuchlicher Ausübung seines Selbstbestimmungsrechtes in Rechte anderer lebensgefährdend eingreift, muss der Staat tätig werden, indem er diesen Menschen entweder in Gewahrsam nimmt, z. B. ins Gefängnis steckt, oder aber ihn unter der Annahme einer krankheitsbedingten Störung (seiner Selbstbestimmungsfähigkeit) in die Psychiatrie bringt. Damit überantwortet der Staat die Lösung des Konflikts zweier einander widersprechender ethischer Prinzipien dem Psychiater, nämlich den Konflikt zwischen der Fürsorgepflicht des Psychiaters und dem Selbstbestimmungsrecht des Patienten. Denn die Fürsorgepflicht, die die Behandlung der die Gefährdung bedingenden psychischen Störung gebietet, wird durch das Selbstbestimmungsrecht des Patienten begrenzt. Dabei geht es im ungünstigsten Fall um die Anwendung von Zwang, den nicht nur der Patient meist ablehnt, sondern auch der Psychiater vermeiden will. Denn auch für die psychiatrisch Tätigen ist Zwangsanwendung immer eine hohe Belastung, sowohl wegen der direkten Anwendung von Gewalt als auch wegen des Wissens um deren traumatisierende Wirkung.

Deshalb soll hier der Blick vor allem auf präventive Strategien zur *Abwendung* von Zwang gerichtet werden und zwar in Kenntnis der Voraussetzungen und Verfahren bei *Anwendung* von Zwang. Zunächst werden

- die ethischen und rechtlichen *Normen* der *Anwendung* von Zwang skizziert, dann
- Schwierigkeiten ihrer Befolgung in der *Praxis* kasuistisch illustriert und schließlich
- der Einfluss des Kontextes deutlich gemacht. Dessen Bedeutung für die *Abwendung*, also die *Prävention* zur Vermeidung von Zwang ist bisher nicht immer genügend berücksichtigt worden, kann aber kaum überschätzt werden.

## Ethische und rechtliche Normen der Anwendung von Zwang

### Definition von Zwang

In den letzten Jahren haben sich normgebende Institutionen wie das Bundesverfassungsgericht [2] und danach der Deutsche Ethikrat [3] mit der Anwendung von Zwang in der Medizin und besonders in der Psychiatrie ausführlich und konkret beschäftigt. Auch die DGPPN (Deutsche Gesellschaft für Psychiatrie und Psychotherapie, Psychosomatik und Nervenheilkunde) [4] hat 2014 und kürzlich mit einer spezifischen S3-Leitlinie [5, 6] zu den Fragen der Anwendung von Zwang detailliert Stellung genommen. Danach werden unter Zwang in seinen ausgeprägtesten Formen

- *Zwangsmaßnahmen* wie „Unterbringungen psychisch Kranker gegen deren erklärten Willen in Krankenhäusern und unterbringungsähnliche Maßnahmen (wie Fixierungen und Isolierungen) gegen den erklärten Willen“ verstanden (also Maßnahmen, die unter dem prototypischen Terminus *„Zwangsunterbringung“* zusammengefasst werden) ebenso wie
- „diagnostische und therapeutische *Interventionen* gegen den erklärten Willen des Patienten“ unter dem prototypischen Begriff der *„Zwangsbehandlung“* zusammengefasst werden.

### Rechtfertigung der Anwendung von Zwang

Solche Anwendungen von Zwang sind nur zu rechtfertigen, wenn

- aus dem Verhalten des Patienten eine schwerwiegende *Gefährdung *seiner selbst (Eigengefährdung) oder anderer Menschen (Fremdgefährdung) – oft in Kombination miteinander – droht und nur durch Zwangsmaßnahmen als Ultima Ratio unter Kontrolle zu bringen ist; und wenn
- dieser Gefährdung eine *Unfähigkeit zu Selbstbestimmung *zugrunde liegt.

Ist eine *Selbstbestimmungsunfähigkeit* nicht zu belegen, dann darf der psychisch kranke Patient nicht gegen seinen Willen behandelt werden. Das führt gelegentlich zu der psychiatrisch unerwünschten Situation, einen psychisch Kranken trotz behandelbarer Krankheit nicht behandeln zu dürfen, aber ihn im Falle einer gerichtlich angeordneten Zwangsunterbringung in Gewahrsam halten zu müssen. Solche ausschließlich fremdnützige Zwangsunterbringung in der Psychiatrie ist ethisch problematisch und konzeptionell weiter untersuchungsbedürftig [7]. Jedoch auch bei *selbstbestimmungsunfähigen* Patienten kann Zwang nur beim Vorliegen der Kriterien der Eigen- oder Fremdgefährdung in Erwägung gezogen werden, während „Maßnahmen gegen den Willen dieser Patienten nicht zu rechtfertigen sind, auch wenn sie aus medizinischer Perspektive zwar indiziert sind, aber im Fall der Ablehnung der Therapie weder für den Betroffenen noch für andere eine erhebliche gesundheitliche Gefahr bedeuten würde. Anstelle von Zwang ist in solchen Fällen ein an den Bedürfnissen und der Lebensrealität der Betroffenen orientiertes Behandlungs- und Unterstützungsangebot vor Ort zu fordern“ [4].

### Selbstbestimmungsunfähigkeit als eine Voraussetzung der Anwendung von Zwang

Es dürfte schon deutlich geworden sein, dass die Wahrnehmung seines Selbstbestimmungsrechtes durch den psychisch Kranken nur dann uneingeschränkt möglich ist, wenn er auch die Fähigkeit dazu hat. Damit gewinnt die Feststellung der Selbstbestimmungsfähigkeit eine zentrale Rolle *vor* der Anwendung von Zwang. Sie gehört zur Kompetenz des Psychiaters, um seine Fürsorgepflicht wahrnehmen zu können, also den psychisch Kranken „vor ungewollten Folgen durch nicht selbstbestimmte“ [4], d. h. vor krankheitsbedingten Entscheidungen zu schützen. Ist das im akuten Notfall nicht möglich, sollte dies wenigstens nach Abwehr der unmittelbaren Gefahr explizit reflektiert und mit möglichst allen Beteiligten besprochen werden.

### Terminologische Zwischenbemerkung

In juristischen Texten, so besonders im Arzneimittelgesetz (AMG), wird als Voraussetzung einer gültigen Einwilligung ein Erfassen vom „Wesen, Bedeutung und Tragweite“ eines Geschehens [8] genannt. Diesem zunächst für die *Forschungsteilnahme *von Patienten entwickelten rechtlichen Ausdruck der *Einwilligungsfähigkeit *ist der übergeordnete Begriff der Selbstbestimmungsfähigkeit vorzuziehen, da er über die Einwilligung („consent“) hinaus auch die Möglichkeit zur Ablehnung („dissent“) anerkennt und zudem deutlich macht, dass hier ein Menschenrecht berührt wird und deshalb die Feststellung der Selbstbestimmungsfähigkeit eine besonders verantwortungsvolle Aufgabe ist [9]. Eine zunehmende Rolle spielt zudem der Begriff des „natürlichen Willens“, mit dem alle intendierten Willensäußerungen des *selbstbestimmungsunfähigen* Kranken, also Willensäußerungen unterhalb der Schwelle zur Selbstbestimmungsfähigkeit gemeint sind.

### Feststellung der Selbstbestimmungsfähigkeit

Die Feststellung der Selbstbestimmungsfähigkeit ist ein eigenes Thema; hier sei nur gesagt, dass die zunächst für die Einwilligung in eine *Forschungsteilnahme* entwickelten Kriterien im Rahmen des Informed-consent-Prozesses auch in der *Praxis*, also mit der für jede ärztliche Intervention erforderlichen Bemühung um Einwilligung des Patienten nach dessen Aufklärung erfasst werden sollen. Dieser Prozess ist noch deutlich optimierbar [10]. Zwar wird das Verständnis der Aufklärung als Voraussetzung einer gültigen Einwilligung bisher meist und wohl zu Recht vorausgesetzt, bedarf aber in fraglichen Fällen und bei psychischen Störungen sorgfältiger Prüfung. Dann reicht es nicht, wenn der Patient zwar schriftlich und dazu auch mündlich Informationen erhält, aber in der Regel nicht gefragt wird, ob er die Informationen auch verstanden hat. Ein solches Verständniskriterium der Einwilligungs- bzw. Selbstbestimmungsfähigkeit ist der sehr allgemeinen juristischen Formulierung des intellektuellen Erfassens vom „Wesen, Bedeutung und Tragweite“ eines Geschehens (AMG) leider nicht zu entnehmen. So wurde sogar im Bundestag u. a. gefragt: „Muss die Information nur gegeben werden oder muss die Informationsgeberin bzw. der Informationsgeber dafür Sorge tragen, dass sie auch verstanden wird?“ [11], obwohl doch klar sein dürfte, dass ohne Verständnis der Informationen, die der Arzt dem Patienten zur Aufklärung zu geben hat, die wesentliche Voraussetzung zur Annahme bestehender Einwilligungsfähigkeit und damit natürlich auch der gültigen Einwilligung entfällt. Auch und besonders dann, wenn ein Patient eine erforderliche oder gar lebensrettende medizinische Maßnahme ablehnt, hat der Arzt die Pflicht, den Patienten umfassend über den Nutzen und die Risiken der Behandlung und die Konsequenzen einer Ablehnung aufzuklären und zu prüfen, ob der Patient die Informationen verstanden hat.

### Anwendungspraxis

Diese Kriterien wurden in den letzten Jahren zu Anweisungen für die Praxis weiterentwickelt. Sie machen die einzelnen Schritte des Prozesses der Aufklärung und Entscheidungsfindung transparent, stellen Kriterien verschiedener Entscheidungsmodelle dar, so von der (1) tradierten paternalistischen Entscheidung des Arztes *für* den Patienten über die (2) von Patient und Arzt *gemeinsam* (partizipativ) gefundene Entscheidung bis zur (3) autonomen Entscheidung *allein* des Patienten, und stärken vor allem im Respekt vor dem Selbstbestimmungsrecht die Möglichkeiten des Patienten zur eigenständigen Entscheidung, gegebenenfalls auch in Form einer Entscheidungsassistenz [12, 13]. Damit wird der Blick von der informierten Einwilligung als einer technischen Pflichterfüllung („empty ethics“ [14]), einer Pflichterfüllung, die eben nicht „zu rechtlicher Formerfüllung verkommen“ [15] darf und damit unzureichend ist, zunehmend auf eine patientenorientierte Zielvorstellung gelenkt. Aber auch dabei sind Risiken zu beachten, wie etwa, dass manche Routineentscheidungen bestimmte Präferenzen des Patienten unberücksichtigt lassen [16, 17] oder aber, dass intensive und affektiv getönte Aufklärungsgespräche das Risiko bergen, die unscharfe Grenze zwischen einem „ethisch zu rechtfertigenden Überzeugungsversuch“ und einer abzulehnenden Manipulation zu überschreiten [4].

## Schwierigkeiten der Befolgung dieser Normen in der Praxis

### Schwierigkeiten in der Feststellung der Selbstbestimmungsfähigkeit

Die Selbstbestimmungsfähigkeit ist keine unveränderliche Eigenschaft des Individuums, sie kann schwanken. Sie unterliegt vielfältigen emotionalen, intentionalen, interpersonellen Einflüssen, der Atmosphäre der aktuellen Situation wie auch biographischen Dispositionen [18]. Ihre Erfassung wird deshalb von der Behindertenrechtskonvention (BRK; engl. CRPD) auch abgelehnt. So kann etwa die kulturelle oder emotionale Abhängigkeit, z. B. von Angehörigen, die Selbstbestimmungsfähigkeit beeinträchtigen. Drei Beispiele:

1. Eine ältere Frau lehnte die Amputation eines Beines ohne Begründung ab, obwohl sie über die tödliche Gefahr durch dessen infizierte Gangrän eingehend informiert worden war. Erst nachdem die Ärzte den Sohn der im Ausland lebenden Italienerin kontaktiert hatten, stimmte sie der Amputation mit der Begründung zu, dass in ihrer Familie immer der älteste Mann die Entscheidungen zu treffen habe.
2. Ein alter Mann fürchtet, die Zuneigung seiner Kinder zu verlieren, wenn er sich gegen deren ihm bekannte Absicht entscheidet, in einem Heim untergebracht zu werden.
3. In der Erregung oder Empörung der abgelehnten Zwangsaufnahme lehnt ein Patient auch die Empfehlung des aufklärenden Arztes rundheraus ab, ohne die ihm dargestellte Notwendigkeit und Nutzen der Intervention zu bewerten bzw. eine rationale Nutzen-Risiko-Bewertung durchzuführen.

Es bedarf nicht selten erheblicher Geduld und Einfühlungsvermögen und gelegentlich auch der Einbeziehung des Umfeldes, von solchen nachvollziehbaren Einschränkungen der Selbstbestimmungsfähigkeit jene Einbußen zu unterscheiden, die durch die psychische Störung, also krankheitsbedingt und anhand definierter psychopathologischer Kriterien zu erfassen sind – denn erstere können durch verstehendes Eingehen auf den Patienten vielleicht aufgelöst und damit Zwang vermieden werden. Aber nicht immer ist diese Unterscheidung möglich, wenn z. B. eine Situation lebensgefährdend zu entgleisen droht und Überlegungen zur Anwendung von Zwang provoziert, ja Zwang unvermeidbar erscheinen lässt. Dabei kann hoher Entscheidungsdruck die Schwelle zur psychiatrischen Feststellung senken, dass die Selbstbestimmungsfähigkeit des Patienten beeinträchtigt oder aufgehoben ist. Als Beispiel weiterer Schwierigkeiten sei nur die Beurteilung erwähnt, ob ein Patient die ihm gegebenen Informationen verstanden hat, wenn er z. B. schweigt oder sich nur vage oder nur fremdsprachlich ausdrücken kann.

### Entscheidung zur Zwangsanwendung

Vor der Anwendung von Zwang ist aber nicht nur zu prüfen, ob 1. die psychischen *Kriterien *der Selbstbestimmungsfähigkeit vorhanden sind (und übrigens auch konkret dokumentiert werden sollten), sondern 2. müssen auch Ausmaß und Konkretheit der *Gefährdung* beurteilt werden. So ist es schon nicht einfach, die Akuität und Ernsthaftigkeit einer Selbsttötungsabsicht zutreffend zu beurteilen, aber gelegentlich ist es noch schwieriger, die Lebensgefährlichkeit einer körperlichen Vernachlässigung einzuschätzen oder auch, inwieweit das Verhalten des psychisch Kranken lebensgefährliche Akte anderer Menschen provozieren kann.

- So sind zur Zeit der Mauer in Berlin psychisch Kranke auf die Mauer geklettert im Wissen darum, dass geschossen werden kann. Nicht immer konnte geklärt werden, ob dahinter Suizidabsichten standen.

Weiterhin muss 3. die Zwangsanwendung indiziert, d. h. geeignet sein, das angestrebte Ziel auch erreichen zu können und schließlich müssen 4. alle weniger eingreifenden Mittel versucht, aber erfolglos geblieben sein.

Was aber sind weniger eingreifende, sog. „mildere“ Mittel? Damit ist das wichtigste Thema angesprochen:

## Vermeidung von Zwang durch präventive Maßnahmen

Eine gute Kenntnis des Patienten ist erforderlich, um z. B. den Hintergrund einer Erregung zu verstehen und in dessen Kenntnis beruhigend – also deeskalierend – mit dem Patienten zu sprechen. Diese Kenntnis ist vornehmlich in – auch vom Gesetzgeber geforderten – Gesprächen mit dem Patienten vorab zu gewinnen, aber auch darüber hinaus mit möglichst allen Betroffenen, also Angehörigen des Patienten und mit dem psychiatrischen Team.

### Vorausverfügungen

Hilfreich ist es, wenn das Ergebnis dieser unverzichtbaren Gespräche schriftlich als *Behandlungsplan *für den Fall einer Krise festgehalten werden kann; in einer solchen Vorausverfügung des selbstbestimmungsfähigen Patienten können vor allem bei rezidivbedrohten Verläufen seine bisherigen positiven wie negativen Erfahrungen mit psychiatrischen Interventionen niedergelegt und in einer kritischen Situation berücksichtigt werden. Breiter gefasst ist eine *psychiatrische Vorausverfügung*: sie enthält Absprachen beispielsweise zur „Einschaltung einer externen Vertrauensperson, zu hilfreichen oder nicht gewünschten Medikamenten, Deeskalationsmaßnahmen vor Zwangsmaßnahmen, ggf. Festlegung der subjektiv am wenigsten belastenden Form von Zwangsmaßnahmen“ [4]; sie können also auch Wertungen, Einstellungen und Präferenzen des Patienten fixieren, die die Entscheidungen der Therapeuten leiten sollen; dabei geht es um das, was der Patient als *sein *Behandlungsziel ansieht, das sich durchaus von dem des Psychiaters unterscheiden kann, z. B. selbständiges Leben trotz weiterbestehender psychopathologischer Symptomatik, etwa im Sinne von „recovery“, auch als „Freiheit zur Krankheit“ [19, 20] beschrieben. Deutlich mehr Patienten sind bereit, eine solche psychiatrische Vorausverfügung bzw. Behandlungsvereinbarung zu verfassen, wenn ihnen dabei beratend geholfen wird: Man spricht dann von *„erleichterter Vorausverfügung“:* „facilitated psychiatric advance directive“ (F-PAD). Kontrollierte Studien haben gezeigt, dass solche Vorausverfügungen die Anwendung von Zwangsmaßnahmen deutlich verringern und das Gefühl der Selbstbestimmung des Patienten stärken. Allerdings ist ihr rechtlicher Status keineswegs einheitlich geregelt; insbesondere bedarf es konzeptueller Klärung und empirischer Überprüfung, in welchen Situationen sie und nach welchen Regeln sie außer Kraft gesetzt werden können, z. B. für Sicherungsmaßnahmen zur unmittelbaren Gefahrenabwehr [21].

### Weitere vertrauensbildende Maßnahmen

Wenn hoher emotionaler Gehalt und Entscheidungsdruck zum Handeln zwingen, dann ist deeskalierende Ruhe und professionelle Besonnenheit notwendig; Möglichkeiten zum Aggressionsabbau, z. B. durch Raum für Bewegung und Präsenz von Personal, sollen vorhanden sein oder geschaffen werden, z. B. durch zureichende Aufstockung von Personal und durch bauliche Veränderungen, deren deeskalierende Wirkung etwa in Tübingen gezeigt werden konnte [22]. Zu vertrauensbildenden Maßnahmen gehört auch, dem Patienten soweit wie möglich Selbstverantwortung zuzugestehen [23], z. B. bei suizidalen Patienten durch engmaschige Verpflichtung per Handschlag oder durch Öffnung der Türen [24, 25]. Gewiss wird die psychiatrische Bereitschaft, damit auch mögliche Risiken einzugehen, individuell unterschiedlich ausgeprägt sein; sie hängt auch vom personalen Umfeld und vom gesellschaftlichen Kontext ab, etwa der schwankenden Ausprägung des öffentlichen Sicherheitsbedürfnisses [26–28].

### Kontexteinflüsse

**Figure 1:**
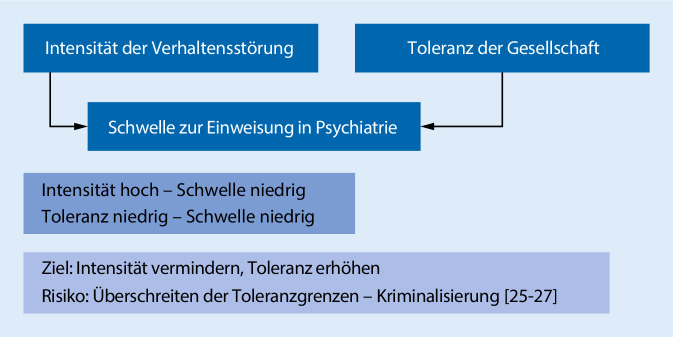

So wurden vielfach erhebliche regionale und epochale Unterschiede in der Häufigkeit von Zwangsanwendungen bei psychisch Kranken festgestellt. Sie weisen darauf hin, dass der Kontext, also Vorstellungen und Erfahrungen der Menschen in der Umgebung des psychisch Kranken und darüber hinaus in der gesellschaftlichen Atmosphäre, etwa im allgemeinen Sicherheitsbedürfnis, in der Häufigkeit und Intensität diskriminierender und stigmatisierender Einstellungen gegenüber psychisch Kranken, die Bereitschaft zur Anwendung von Zwang erheblich beeinflusst (Abb. 1).

Diese Einstellungen und Vorurteile haben sich über lange Zeit entwickelt: Noch vor 150 Jahren fand beispielsweise ein Arzt im Schweizer Kanton Fribourg mit Stricken gefesselte und in Tierställe oder gar in einen Backofen weggesperrte Menschen, gegen deren ausgeprägte Verhaltensstörung sich ihre Angehörigen offenbar nicht anders zu helfen wussten [29]; vor 100 Jahren propagierte die eugenische Bewegung die Prävention psychischer Krankheiten und Behinderung durch Sterilisation, und nationalsozialistische Propaganda stellte psychisch Kranke als „nutzlose Esser“ dar [30]; vor 50 Jahren begannen Psychiater gegen die „brutale Wirklichkeit“ der nach dem Krieg völlig heruntergekommenen psychiatrischen Krankenhäuser so heftig zu protestieren, dass die Politik erreicht und mit der Psychiatrie-Enquète des Bundestages eine tiefgreifende Verbesserung der Versorgung psychisch Kranker erreicht wurde [31]. Aber diese Vergangenheit hat im Bewusstsein der Menschen Spuren hinterlassen, die auch heute noch wirksam sind [32]. Der britische Psychiater George Szmukler hat wohl Recht, wenn er sagt „It would be foolish to think that such long history of profound discrimination and stigmatization can be easily undone“ [33].

### Sensibilisierung gegenüber Zwang

Der Psychiater hat gelernt, in der Beziehung zum psychisch Kranken seine eigenen Gefühle und Einstellungen zu reflektieren und zu kontrollieren. Denn Gefühle von Sympathie oder Antipathie können in sein Verhalten ebenso einfließen wie Einstellungen seiner Umgebung und im weiteren Sinne auch der gesellschaftlichen Atmosphäre. Ich will dies mit zwei Beispielen kurz andeuten, eines für unmittelbare Einflüsse auf der interpersonell-individuellen Ebene, ein zweites für Einflüsse aus dem gesellschaftlichen Umfeld:

1. Innerhalb einer „Pyramide der Ausübung von Druck“ [33] gegen zögernde oder ablehnende Patienten verschwimmt oft die Grenze zwischen „Überzeugung, Überredung, Anreiz, gefühlsmäßiger Beeinflussung, Drohung“; so wurde früher wohl häufiger gegenüber erheblich verhaltensgestörten und behandlungsbedürftigen Patienten argumentiert „Wenn Sie die Medikation nicht nehmen, wird ein Richter Sie zwangseinweisen müssen“; heute allerdings ist die darin auch liegende Drohung den meisten psychiatrisch Tätigen bewusst. Jede Entscheidung dieser Alternativen wird der Patient als Zwang erleben. Dieses Risiko ist aber vielleicht zu vermeiden, wenn die Behandlungsnotwendigkeit dem Patienten als Angebot offeriert wird, so etwa – nach einem Vorschlag von Szmukler – „Wenn sie die Medikation nehmen, dann können Sie mit anderen Patienten zusammen Ausgang haben.“ [33].
2. Mein zweites Beispiel: Die in der damaligen sowjetischen Psychiatrie benutzte Diagnose der „schleichenden Schizophrenie“ vermittelte – in nach den heute üblichen diagnostischen Kriterien höchst fraglichen Fällen – den Eindruck einer eindeutigen Krankheit und konnte zusammen mit den weiteren Annahmen, dass die inkriminierten abweichenden Verhaltensweisen Ausdruck psychischer Krankheit sind, die in der Regel zur *Schuldunfähigkeit* führt, Zwangsmaßnahmen begründen. Die letztgenannte Annahme wurde dem Psychiater in einer Gesellschaft sehr nahegelegt, deren Verfassung den Bürger „verpflichtet, die Interessen des Sowjetstaates zu schützen und zur Stärkung seiner Macht und seiner Autorität beizutragen“ und in der folgende – vom damaligen sowjetischen Staatschef Nikita Chruschtschow formulierte – Norm herrschte: „Ein Verbrechen ist eine Abweichung von den allgemein anerkannten Standards des Verhaltens, das oft durch psychiatrische Krankheit verursacht wird.“ [34].

Diese Empfindlichkeit gegen öffentliche Normabweichungen führte zur Akzeptanz von Zwang, etwa Zwangsunterbringungen von Menschen mit psychischen Krankheiten, die in anderen Ländern ohne Zwang behandelt worden wären.

## Schlussfolgerungen

Das Wissen, dass und wie welche Einflüsse die Selbstbestimmungsfähigkeit des psychisch Kranken und deren Erfassung beeinflussen können, gehört ebenso zur Kompetenz des Psychiaters wie die Einschätzung aktueller Gefährdungen, damit er seiner Fürsorgepflicht nachkommen kann. Deshalb sollen der Psychiater (und seine Mitarbeiter im Rahmen ihrer Verantwortlichkeit) geschult sein:

1. In der *Feststellung der Selbstbestimmungsfähigkeit*, die bei Vorhandensein jeden Zwang zur ärztlichen Intervention gegen den erklärten Willen des Patienten ausschließt.
2. In der *Risikoabschätzung* einer Zwangsanwendung, d. h. der Berücksichtigung des „Nicht-Schadens-Prinzips“; sie kann erhebliche Anforderungen stellen, etwa bei der Frage, wie erheblich und wie konkret eine aktuelle Gefährdung tatsächlich ist oder „wie schwerwiegend und wie wahrscheinlich Konsequenzen der Nichtbehandlung tatsächlich sein müssen, um eine Maßnahme gegen den ‚natürlichen‘ Willen, d. h. gegen eine aktuelle ablehnende Willensäußerung des *selbstbestimmungsunfähigen* Patienten, durchsetzen zu dürfen“ [4].
3. Und vor allem sollen er und sein professionelles Team geübt sein in der *präventiven Abwendung von Zwang*, um gefährdetes Leben auch ohne Zwang schützen und dem Selbstbestimmungsrecht patientenorientiert weitgehend Rechnung tragen zu können.
4. Qualifizierende *Weiterbildung* mit supervidierten Fallbeispielen in Deeskalations- und Abwehrtechniken zu ihrer Umsetzung in der Praxis sind in den letzten Jahren bereits in vielen psychiatrischen Institutionen eingeführt worden; sie ist aber hinsichtlich der rechtlichen und ethischen Normen psychiatrischen Handelns dringend weiter auszubauen. Denn Psychiater und ihre Teams sind sich zwar bewusst, dass die Anwendung von Zwang ethische Fragen aufwirft. Oft findet sich jedoch eine Unsicherheit dabei, die implizierten ethischen Fragen klar zu formulieren und die Anwendung von Zwang argumentativ ethisch zu begründen [35].
5. Zeitarmut höhlt ethische und rechtliche Vorgaben aus, fördert strukturelle Gewalt, aktiviert antipsychiatrische Kritik und fördert Stigmatisierung nicht nur des psychisch kranken Individuums, sondern auch der Institution Psychiatrie und der in ihr Arbeitenden, wenn unverzichtbare und rechtlich geforderte Gespräche infolge Zeitmangels nur unzureichend möglich sind: also
  Gespräche mit dem Patienten zur Vertrauensbildung, zur Deeskalation, insbesondere auch zur Hilfe für eine Vorausverfügung sowie zur Feststellung der Selbstbestimmungsfähigkeit vor der Anwendung von Zwang und
  Gespräche im psychiatrischen Team und mit Angehörigen zur Abschätzung der Risiken der Anwendung von Zwang sowie
  Zeit für die qualifizierte Weiterbildung von Kompetenz zur Prävention, d. h. zur Abwendung von Zwang.
    Träger psychiatrischer Institutionen müssen deshalb die *Rahmenbedingungen psychiatrischen Handelns* so gestalten, dass den Mitarbeitern die Zeit zur Verfügung steht [36], um diese Gespräche – oft mit viel Geduld und in notwendiger Ausführlichkeit – tatsächlich führen zu können. Dass hier erheblicher Handlungsbedarf besteht, macht die Feststellung der DGPPN deutlich: „Wenn die Spannung zwischen Anforderungen des BGB und unzureichender Ausstattung durch das Sozialrecht weiter zunehmen, wird die Gesetzgebung unglaubwürdig und die im Gesundheitswesen Tätigen kommen in eine ethisch nicht zu verantwortende Konfliktsituation“ [4].
    Konzeptuelle und empirische *Forschung* ist erforderlich, um etwa Kriterien für die Abschätzung von Gefährdungen oder für die Reichweite von Vorausverfügungen weiterzuentwickeln und um die Wirksamkeit der vorgetragenen Maßnahmen in der psychiatrischen Praxis zu überprüfen. Denn bisher ist die Evidenz vieler Maßnahmen verbesserungsbedürftig.
6. Die *Zielvorstellung* einer psychiatrischen Behandlung ohne Anwendung von Zwang ist am *Selbstbestimmungsrecht* des psychisch Kranken orientiert. Kürzlich wurde das Konzept einer Psychiatrie „Ohne Zwang – für eine ausschließlich unterstützende Psychiatrie“ publiziert [37]. Diese radikale Interpretation der UN-Behindertenrechtskonvention will die Psychiatrie auf eine psychosoziale Hilfspflicht begrenzen und jegliche ordnungspolitisch begründete Maßnahme in die ausschließliche Zuständigkeit der Polizei und Justiz verweisen. Das mag manchem wünschenswert erscheinen, ist jedoch mit unserer gegenwärtigen, am Konzept der *Selbstbestimmungsfähigkeit* orientierten Rechtslage nicht vereinbar und müsste grundlegende Schwierigkeiten in rechtlicher, aber auch in ethischer und praktischer Hinsicht überwinden [38]. Überdies geht diese Konzeption einer ausschließlich Willen und Präferenzen des psychisch Kranken unterstützenden, und damit das Konzept der Selbstbestimmungsfähigkeit ablehnenden Psychiatrie vor allem an der Hilfsbedürftigkeit *selbstbestimmungsunfähiger* psychisch Kranker vorbei, man denke nicht nur an Wahnkranke, deren Wahn sie zu lebensgefährlichen Handlungen zwingt, sondern auch an demente oder delirante Menschen, die in ihrer Verwirrtheit Hilfe gar nicht aufsuchen können, zu deren lebenserhaltender Hilfe der Psychiater aber verpflichtet ist. Wenn dabei Zwang unvermeidbar ist, dann ist er nach Maßgabe der gegenwärtig in Gang gekommenen Entwicklungen wie
  zum einen: Einstellungswandel hin zur Entscheidungsassistenz,
  zum anderen: Präzisierung von Indikation und Kontrolle der Anwendung von Zwang
  und vor allem: Prävention der *Anwendung*, also *Abwendung* von Zwang einzusetzen. Ziel muss sein, diese Entwicklung fortzuschreiben und in der Praxis voll zur Geltung zu bringen.

## Footnotes

1 Art 1(2) Das Deutsche Volk bekennt sich darum zu unverletzlichen und unveräußerlichen Menschenrechten als Grundlage jeder menschlichen Gemeinschaft, des Friedens und der Gerechtigkeit in der Welt. Art 2(1) Jeder hat das Recht auf die freie Entfaltung seiner Persönlichkeit, soweit er nicht die Rechte anderer verletzt und nicht gegen die verfassungsmäßige Ordnung oder das Sittengesetz verstößt. Art 2(2) Jeder hat das Recht auf Leben und körperliche Unversehrtheit. Die Freiheit der Person ist unverletzlich. In diese Rechte darf nur auf Grund eines Gesetzes eingegriffen werden.
